# Children with obesity have poorer circadian health as assessed by a global circadian health score

**DOI:** 10.1007/s12519-024-00804-3

**Published:** 2024-06-08

**Authors:** María Rodríguez-Martín, Nuria Martínez-Lozano, Vicente Santaclara-Maneiro, Antonio Gris-Peñas, Diego Salmerón, Rafael Ríos, Asta Tvarijonaviciute, Marta Garaulet

**Affiliations:** 1 Department of Physiology, Regional Campus of International Excellence, University of Murcia, 30100 Murcia, CP Spain; 2 Biomedical Research Institute of Murcia, IMIB-Arrixaca-UMU, University Clinical Hospital, 30120 Murcia, Spain; 3 Health Area of Lorca, Lorca, Murcia Spain; 4 Health and Social Sciences Department, University of Murcia, Murcia, Spain; 5 Consortium for Biomedical Research in Epidemiology and Public Health, Madrid, Spain; 6 Interdisciplinary Laboratory of Clinical Analysis (Interlab-UMU), University of Murcia, Murcia, Spain; 7 Division of Sleep and Circadian Disorders, Brigham and Women’s Hospital (BWH), and Division of Sleep Medicine, Harvard Medical School, Boston, MA 02115 USA

**Keywords:** Children, Chronodisruption, Circadian, Lifestyle, Obesity

## Abstract

**Background:**

Circadian health refers to individuals’ well-being and balance in terms of their circadian rhythm. It is influenced by external cues. In adults, a close relationship between circadian-related alterations and obesity has been described. However, studies in children are scarce, and circadian health and its association with obesity have not been evaluated globally. We aimed to assess whether circadian health differed between children with and without obesity as determined by a global circadian score (GCS) in a school-age population.

**Methods:**

Four hundred and thirty-two children (7–12 years) were recruited in Spain. Non-invasive tools were used to calculate the GCS: (1) 7-day rhythm of wrist temperature (T), activity (A), position (P), an integrative variable that combines T, A, and P (TAP); (2) cortisol; and (3) 7-day food and sleep records. Body mass index, body fat percentage, waist circumference (WC), melatonin concentration, and cardiometabolic marker levels were determined.

**Results:**

Circadian health, as assessed by the GCS, differed among children with obesity, overweight, and normal weight, with poorer circadian health among children with obesity. Children with obesity and abdominal obesity had 3.54 and 2.39 greater odds of having poor circadian health, respectively, than did those with normal weight or low WC. The percentage of rhythmicity, a marker of the robustness of the TAP rhythm, and the amplitude, both components of the GCS, decreased with increasing obesity. Different lifestyle behaviors were involved in the association between circadian health and obesity, particularly protein intake (*P* = 0.024), physical activity level (*P* = 0.076) and chronotype (*P* = 0.029).

**Conclusions:**

The GCS can capture the relationship between circadian health and obesity in school-age children. Protein intake, physical activity level, and chronotype were involved in this association. Early intervention based on improving circadian health may help to prevent childhood obesity.

**Supplementary Information:**

The online version contains supplementary material available at 10.1007/s12519-024-00804-3.

## Introduction

Childhood obesity is a significant public health concern [1, 2]. Nevertheless, successful treatments for obesity in children are challenging [3], and the underlying causes of overweight at early ages should be investigated to improve the prevention and treatment of obesity in children. Chronobiology is an emerging science that studies the relationship between circadian rhythm alterations, chronodisruption, and pathology [4]. Recent studies link energy regulation to the circadian clock at the behavioral, physiological, and molecular levels [5, 6]. Circadian health refers to the well-being and balance of an individual’s circadian rhythm. It is influenced by external cues, most notably the light–dark cycle of day and night, but other behaviors, such as sleep/wake, eating/fasting, rest/activity, posture changes, and exercise, are also relevant factors [7].

We and others have shown that is more difficult in children with obesity than in those without obesity to maintain healthy biological rhythms (i.e., daily metabolic cycles in humans such as sleep–wake cycles, hormone release, eating habits and digestion, body temperature, and other functions repeated daily) [8]. Circadian-related behaviors such as late bedtimes and late eating have been associated with greater adiposity and obesity in children [9, 10]. However, studies on circadian health in school-age children are still scarce. Its relationship with obesity, including endogenous, behavioral, and environmental markers of circadian health, has not been evaluated globally.

Previously, we developed a global circadian score (GCS) that has been demonstrated to be reliable for assessing circadian health in children [11]. The score includes the following: (1) circadian-related markers such as the amplitude of the rhythm and rhythmicity characteristics of an integrative variable TAP, which combines wrist temperature (T) [12], physical activity (A) [13], and body position (P) [14]; (2) food timing, particularly breakfast and dinner timing [10]; and (3) endogenous circadian-related markers such as cortisol [15].

The hypothesis is that children with poor circadian health are more predisposed to suffer from obesity (and vice versa). Poor circadian health is considered a state in which children’s circadian rhythms are disrupted or not optimally aligned with the natural light–dark cycle [7]. The present study aimed to determine the potential association between circadian health and obesity in school-age children using a global marker of circadian health (GCS). We will also test the independent associations of several circadian-related features involved in the GCS with obesity.

## Methods

### Subjects

Healthy children (7–12 years; *n* = 432) from three schools in a Mediterranean area of Spain were recruited between October 2014 and June 2016 (ClinicalTrials.gov ID: NCT02895282), as previously described [11]. Approval for this study was obtained from the University of Murcia Ethics Committee (ID: 1868/2018). All procedures followed the ethical standards of the institutional and national research committees and complied with the 1964 Helsinki Declaration and its later amendments or comparable ethical standards. The present study is a secondary analysis of the same cohort of children studied in our previous works [10, 11, 16–18].

### Obesity-related trait measurements

Body weight, body fat percentage, and waist circumference (WC) were measured in the whole population (*n* = 432) on the first day of the week of the study and at the same time in the morning, as we previously published [11]. Body weight was measured in barefooted children wearing light clothes using a digital scale accurate to the nearest 0.1 kg. Height was determined using a portable stadiometer (rank: 0.14–2.10 m). The children were positioned upright, relaxed, and with their heads in the Frankfort plane. These data were used to calculate body mass index (BMI) according to the formula weight (kg)/height^2^ (m^2^). BMI was further transformed to the BMI *Z*-score according to the World Health Organization growth reference [19]. Total body fat was determined by bioelectrical impedance using TANITA TBF-300 equipment (Tanita Corporation of America, Arlington Heights, IL) [11]. Several cardiometabolic traits, such as glucose, triglyceride, and cholesterol levels, and inflammatory markers such as C-reactive protein, immunoglobulin A, interleukin (IL)-8, IL-1b, tumor necrosis factor-alpha and monocyte chemotactic protein 1 were determined from serum samples obtained from a subset of 79 children in the morning under fasting conditions as previously described [11].

### Global circadian score determination

An already developed formula was used to determine the GCS for each child [11] (Supplementary Table 1). The score for each factor was calculated by multiplying each variable by its eigenvalue. Then, each factor score was multiplied by the percentage of the variance in the total information explained by that factor. Factors 1 and 2 explained 28% of the variance and were loaded mainly by TAP characteristics, factor 3 explained about 10% (loaded by cortisol measures), and factors 4 and 5 together explained about 13% of the variance and loaded on the timing of food intake (breakfast and dinner). The five factors together explained 50% of the variance in total information.

### Factors and variables included in the GCS

A description of each variable included in the formula is presented in Table 1. Factors 1 and 2 from the GCS included variables derived from TAP. To determine TAP, children wore a wristwatch in their non-dominant hands during the seven days of the study. This wristwatch integrates two sensors: a temperature sensor [20] (Thermochron iButton DS1921H, Dallas, Maxim, Dallas, TX, USA), which is programmed to collect information every 5 minutes; and an accelerometer sensor (G Acceleration Data Logger UA-004–64; Onset Computer, Bourne, MA, USA), which measures physical activity and body position rhythms to record data every 30 seconds [14]. The position was expressed in degrees (°), and activity was expressed as the change in degrees per minute (Δ°/minute). TAP was calculated as previously described [14]. From TAP, different parameters were derived (Table 1) using an integrated package for temporal series analysis (Kronowizard; https://kronowizard.um.es/kronowizard; Chronobiology Laboratory, University of Murcia, Murcia, Spain, 2015). Factor 3 from the GCS comprised salivary cortisol measurements. Saliva samples were collected on the same day at three different times using salivates (Sarstedt, Barcelona, Spain): before breakfast (9:00), before lunch (14:00), and after dinner (23:00). The saliva concentration of cortisol was determined by radioimmunoassay (IZASA, Barcelona, Spain). Factors 4 and 5 from the GCS included dinner start time and finish time and breakfast start time and finish time determined by 7-day dietary records completed by children and parents. Melatonin and lunch timing were not included in the GCS because, in the factor analysis performed for developing the GCS, melatonin and lunch timing did not appear within the first five factors that accounted for about 50% of the variance.

**Table 1:** Description of each variable included in the global circadian score formula

Term (abbreviation and units)	Definition	Type of analysis
Factors 1 and 2		
TAP (arbitrary units)	An integrative variable combining temperature (T), activity (A) and position (P)	
Circadian function index (CFI; range: 0–1)	A numerical index that determines the circadian robustness, based on three circadian parameters: IS, IV and RA. CFI oscillates between 0 (absence of circadian rhythmicity) and 1 (a robust circadian rhythm)	Non-parametric
Amplitude	Difference between the peak (or nadir) and the mean value of a wave	Cosinor
Percentage of rhythmicity (PR; range: 0–100)	Percentage of data variance explained by the sinusoidal function. Higher values of this parameter mean a more sinusoidal curve	Cosinor
Interdaily stability (IS; range: 0–1)	Determines the constancy of the 24 h rhythmic pattern over days. A stable rhythm is characterized by a 24-h profile that remains very similar from one day to another	Non-parametric
Intraday variability (IV; range: 0–2)^a^	This variable is included in CFI and characterizes rhythm fragmentation. Its values oscillate between 0 when the wave is perfectly sinusoidal and 2 when the wave describes a Gaussian noise	Non-parametric
Relative amplitude (RA; range: 0–1)	Difference between the maximum (or minimum) value of the cosine function and mesor. It is calculated by the difference between the average of measurements for the ten consecutive hours with the maximum values (M10) and the average of measurements made for the five consecutive hours with the minimum values (L5), divided by the sum of both values (M10 + L5)	Non-parametric
Mesor	Mean value of the rhythm fitted to a cosine function	Cosinor
Average	Average of the TAP values	Non-parametric
Factor 3		
Cortisol 23:00 (nmol/L)	Salivary cortisol concentration	
Log morning/evening cortisol ratio	Salivary cortisol concentration	
Cortisol 14:00 (nmol/L)	Salivary cortisol concentration	
Factors 4 and 5		
Dinner start time (h)	Determined by 7-d dietary records completed by children and parents	
Dinner finish time (h)	Determined by 7-d dietary records completed by children and parents	
Breakfast start time (h)	Determined by 7-d dietary records completed by children and parents	
Breakfast finish time (h)	Determined by 7-d dietary records completed by children and parents	

^a^IV is included in CFI

### Lifestyle variables

#### Sleep characteristics, individual chronotype, and related variables

We determined sleep timing, sleep duration, and daytime napping timing and duration using 7-day sleep diaries; children and parents recorded the wake/sleep cycle, which has been demonstrated to be a convenient tool for assessing sleep quality and duration [15, 21]. Individual chronotypes were subjectively determined using an age-appropriate Spanish version of the Munich ChronoType Questionnaire (MCTQ) [22], while melatonin was used as an objective proxy of individual chronotypes. Melatonin concentrations were determined via radioimmunoassay (IBL, Germany) in saliva samples collected at night (01:00) and before lunch (14:00). Social jet lag*,* which represents different sleep schedule behaviors between free days and schooldays, was measured as the difference between mid-sleep on free days and mid-sleep on schooldays. We considered that children had social jet lag when there was more than a two-hour difference in the midpoint of sleep between free days and school days [23].

#### Food intake

Energy and macronutrient distributions across meals and the percentage of energy provided by macronutrients were determined by 7-day food diaries adapted for this age group [24], which specify the timing of food intake, type of food and amount/weight/quantity of food eaten [15]. Total energy intake and macronutrient composition were analyzed with a nutritional evaluation software program (Grunumur 2.0 8) [25] based on Spanish food composition tables [26]. Total morning intake was defined as the sum of every intake in the morning, including lunch. Every intake after lunch, including dinner, was considered total evening intake. The timing of meals was determined by averaging every meal timing during the seven days of the dietary records completed by the children and parents.

#### Physical activity

The average physical activity was determined by an accelerometer (G Acceleration Data Logger UA-004–64; Onset Computer, Bourne, Massachusetts, USA) and was calculated as the average accumulative degree change in three-axis tilt per minute during the most active ten hours during the wake period (i.e., nonsleep time), which correlates with motor activity measured by the wrist-worn Actiwatch [11, 27]. We classified physical activity levels by dividing the average physical activity into tertiles (low, medium and high). The day–night physical activity contrast was determined as the relative amplitude of the activity rhythm.

#### Light exposure

We evaluated light exposure near the face (eyes) via a pendant luxmeter in the neck (HOBO Pendant Light Data Logger, UA-002–64; Onset Computer, Bourne, Massachusetts, USA). The instrument was programmed to collect light information continuously every 30 seconds for 7 days. We calculated the lux logarithm collected every 30 seconds to assess the light average. We further calculated the amplitude, percentage of rhythmicity (PR), interdaily stability (IS), and circadian function index (CFI) of the light data rhythm. The description and calculation of these variables are included in Table 1. Children were instructed to wear the pendant over their clothing. When sleeping, they had to leave it on the bedside table. Cortisol and melatonin salivary determinations were performed on Sundays after the 7-day recording of temperature, activity, position, and light was completed to avoid these determinations being affected by the melatonin sampling.

### Statistical methods

Differences in GCS were analyzed among children stratified into groups according to three categories of obesity (children with normal weight, overweight and obesity) according to the sex- and age-specific BMI cutoff points proposed by the International Obesity Task Force [28] via ANCOVAs. When significant, we explored differences among groups using post hoc analysis (Bonferroni). The median GCS was used as a cutoff point for classifying children according to good or poor circadian health. We fitted multinomial logistic regression models to estimate the odds ratios and 95% confidence intervals of having poorer circadian health (as an outcome) in the presence of obesity/overweight (combination of overweight and obesity categories [28]) or high WC (divided according to the median, considering as "low WC" a WC lower or equal to 64 cm, and "high WC" a WC greater than 64 cm).

We tested for linear regression models of the GCS and its components with BMI, BMI *Z*-score, and body fat percentage. To understand the impact of lifestyle on the association between GCS and obesity, we explored the significant interactions between the GCS and lifestyle factors (already identified) and BMI. To identify those lifestyle factors, we previously tested linear regression models to determine whether there were significant associations between GCS and lifestyle factors or between the GCS and obesity traits (BMI, BMI *Z*-score, and body fat percentage). Because no significant interaction effect was found between sex and GCS for BMI, we performed statistical analyses of the total number of children studied without separating by sex. All analyses were performed using SPSS version 25.0 (SPSS, Chicago, Illinois, USA). *P* < 0.05 was considered to indicate statistical significance and is presented in the tables and figures in bold. The results of borderline statistical significance (considering those with *P* < 0.1) are presented in italics in all tables and figures.

We adjusted all analyses, including ANCOVA, multinomial logistic regression, linear regression, and interactions according to sex, age, race, and academic year. We chose those variables as a way to control for sociodemographic status.

## Results

### General characteristics, global circadian score, and obesity traits

**Fig. 1:**
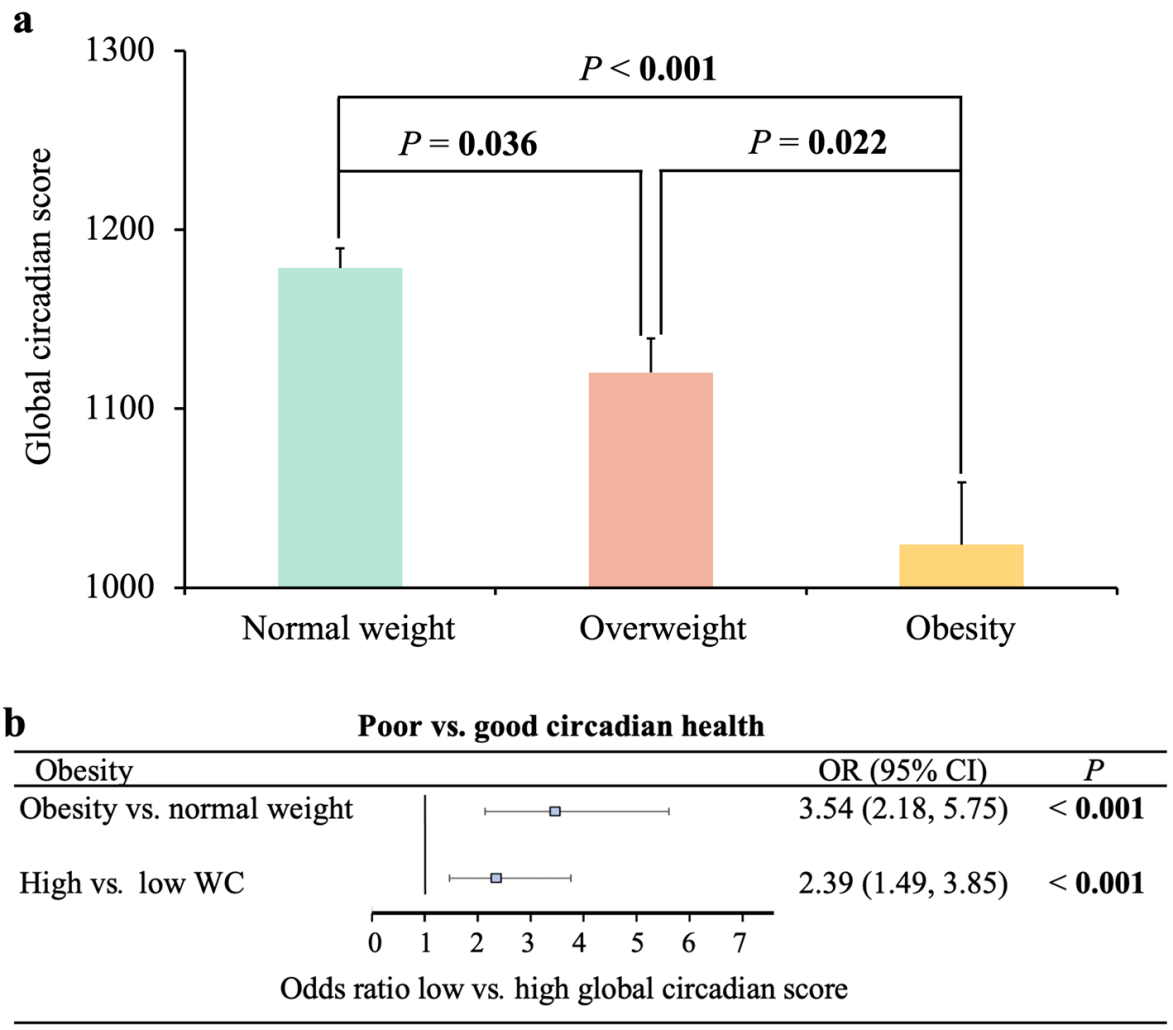
Circadian health and obesity in children. **a** Differences in the global circadian score among children with normal weight (*n* = 268), overweight (*n* = 83), and obesity (*n* = 39) individuals classified according to International Obesity Task Force body mass index (BMI) cutoff points [28]. ANCOVA was performed, adjusting for sex, age, race, and academic year (*P* < 0.001). Significant differences were indicated between each pair of groups according to Bonferroni post hoc analyses (*P* = 0.036, normal weight vs. overweight; *P* < 0.001, normal weight vs. obesity; *P* = 0.022, overweight vs. obesity); **b** odds ratio (OR) and 95% confidence interval (CI) of poor vs. good circadian health with obesity. Obesity, in this case, is a combination of obesity and overweight according to the sex-and-age-specific BMI cutoff points proposed by the International Obesity Task Force [28]; waist circumference (WC) was classified according to the median, considering high WC when WC was greater than 64 cm (and low WC when WC was ≤ 64 cm). All analyses were adjusted for sex, age, race, and academic year. The values in bold are highlighted as statistically significant (*P* < 0.05)

The GCS ranged from 385 to 1555, with higher values corresponding to better circadian health. Descriptive data on the GCS, obesity traits, and related lifestyle factors of the children studied are shown in Table 2. When children were classified into three categories according to their BMI (with obesity, with overweight, and with normal weight), the GCS differed across the three groups toward poorer circadian health among the children with obesity (Fig. 1a). We further classified children into two categories according to their GCS: poor circadian health, with a GCS lower than 1184.61; and good circadian health, with a GCS of 1184.61 or higher. Those children with obesity (determined according to the sex- and age-specific BMI cutoff points proposed by the International Obesity Task Force [28]) and with abdominal obesity (with high WC, if WC > 64 cm, classified according to the median) had 3.54 and 2.39 greater odds of having poor circadian health than those with normal weight [28] or with low WC (WC ≤ 64 cm), respectively (after adjusting for sex, age, race and academic year) (Fig. 1b). Secondary linear regression analyses between the GCS and several obesity-related inflammatory markers showed that the GCS was inversely correlated with C-reactive protein (a trend) (beta = -0.004 ng/mL; *P* = 0.058), while no significant associations were detected with the other inflammatory markers tested [immunoglobulin A, interleukin (IL)-8, IL-1b, tumor necrosis factor-alpha and monocyte chemotactic protein 1; *P* > 0.1].

**Table 2:** Descriptive data of the total children studied

Variables	*n*	Mean (SD)
Girls/boys^a^, %	219/213	50.7/49.3^a^
Age (y)	432	10.13 (1.25)
Race^a^, %caucasian	432	93.3^a^
Academic year^a^, %6th primary school year	432	40.5^a^
Obesity traits		
Weight (kg)	431	41.42 (12.24)
Height (cm)	431	145.05 (10.37)
BMI (kg/m^2^)	431	19.36 (3.95)
BMI *Z*-score	411	1.26 (2.26)
Obesity degree		
Normalweight^a^, %	289	67.05^a^
Overweight^a^, %	97	22.51^a^
Obesity^a^, %	45	10.44^a^
Body fat, %	347	21.19 (7.53)
Waist (cm)	352	65.44 (9.89)
Glucose (mg/dL)	79	83.44 (9.13)
Triglycerides (mg/dL)	78	80.50 (30.89)
Cholesterol (mg/dL)	79	169.04 (33.75)
Sleep characteristics		
Bedtime (h)	401	22.82 (0.64)
Awake time (h)	401	8.21 (0.48)
Nighttime sleep duration (h)	401	9.33 (0.66)
Number of awakenings during the nighttime sleep	229	0.045 (0.11)
Siesta yes/no^a^, %	53/379	12.27/87.73^a^
Siesta start time (h)	53	16.35 (0.81)
Siesta duration (min)	53	1.06 (0.62)
Social jet lag (h)	414	1.35 (0.70)
Chronotype (MCTQ score)	419	4.04 (0.67)
Melatonin 01:00 (pg/mL)	413	26.31 (17.72)
Melatonin 14:00 (pg/mL)	409	7.31 (5.87)
Difference in melatonin night-morning	407	19.43 (16.43)
Food intake characteristics		
Lunch start time^b^ (h)	405	14.39 (0.32)^b^
Lunch finish time^b^ (h)	405	14.93 (0.37)^b^
Total daily energy intake (kcal)	379	2015.72 (412.64)
Total daily energy intake (kJ)	379	8433.77 (1726.49)
Total morning intake^c^ (kcal)	374	1207.13 (239.43)^c^
Total morning intake^c^ (kJ)	374	5050.63 (1001.78)^c^
Total evening intake^c^ (kcal)	376	812.67 (244.72)^c^
Total evening intake^c^ (kJ)	376	3400.21 (1023.91)^c^
Energy intake distribution by meals		
Breakfast intake, % of total kcal	379	17.25 (6.41)
Midday snack intake, % of total kcal	374	10.57 (4.23)
Lunch intake, % of total kcal	379	32.62 (5.96)
Afternoon snack intake, % of total kcal	376	12.05 (5.50)
Dinner intake, % of total kcal	379	27.73 (6.36)
Macronutrient distribution		
Carbohydrate intake, % of total kcal	379	43.45 (5.65)
Lipid intake, % of total kcal	378	44.26 (6.36)
Protein intake, % of total kcal	379	14.33 (2.13)
Physical activity level		
Physical activity average (Δº/min)	412	46.46 (6.09)
Day-night physical activity contrast (RA)	412	0.84 (0.05)
Light exposure		
Amplitude (log lux)	120	1.41 (0.17)
PR	120	49.99 (7.43)
IS	120	0.66 (0.08)
CFI	120	0.86 (0.03)
M10 (log lux)	120	2.30 (0.24)
Day-night light contrast (RA)	120	1.00 (0.01)
Global circadian score	391	1150.23 (189.62)

Mean (SD) of descriptive variables of the children studied. *GCS* global circadian score, *BMI* body mass index (kg/m^2^), *MCTQ* Munich chronotype questionnaire (score < 3: morning type, score 3-5: intermediate type, score > 5: evening type), *kcal* kilocalories, *kJ* kilojoules, *PR* percentage of rhythmicity, *IS* interdaily stability, *CFI* circadian function index, *M10* average of measurements of the 10 consecutive hours with the maximum light exposure (log lux), *RA* relative amplitude, *SD* standard deviation. ^a^Frequency for categorical variables (%); ^b^lunch timing not included in GCS; ^c^total morning intake calculated as: breakfast + midday + lunch energy intake, and total evening intake as afternoon + dinner energy intake

In addition, several variables included in the GCS were independently associated with BMI and body fat percentage **(**Table 3**)**. For the integrated TAP variable, the PR, amplitude, IS, CFI, and other circadian-related variables were inversely associated with BMI and BMI *Z*-score (*P* < 0.05). Cortisol level at 14:00 was also inversely associated with BMI and body fat percentage. In contrast, dinner start and finish time were directly associated with body fat percentage (toward later timing with greater body fat) **(**Table 3**).**

**Table 3:**
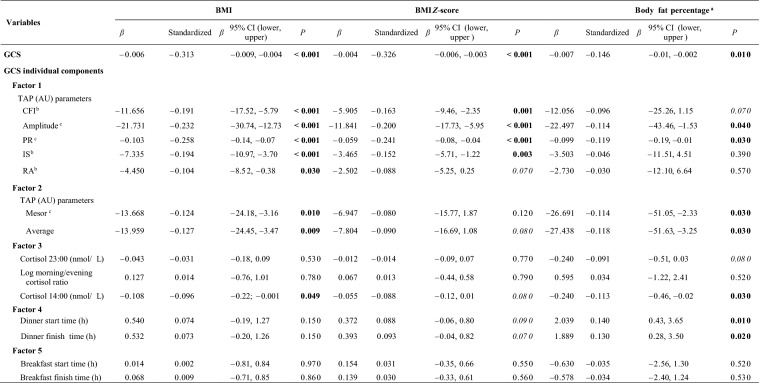
Association of GCS and its individual components with BMI, BMI *Z*-score and body fat percentage

Linear regression of GCS (independent variable) (and its individual components with each factor ordered as it appears in the formula) with BMI/BMI *Z*-score or body fat percentage as dependent variables, adjusting by sex, age, race, and academic year. *P* values in bold indicates statistically significant differences (*P* < 0.05); in italic indicates *P* < 0.1. *GCS* global circadian score, *BMI* body mass index, *CI* confidence interval, *TAP* integrative variable combining temperature, activity and position, *AU* arbitrary units, *CFI* circadian function index, *PR* percentage of rhythmicity, *IS* interdaily stability, *RA* relative amplitude. ^a^*n* = 346; ^b^parameters derived from non-parametric analyses; ^c^parameters derived from Cosinor analyses

### The role of lifestyle behaviors

Lifestyle factors information significantly associated with circadian health and obesity traits is presented in Table 4. Interestingly, earlier bedtime and awake time, longer duration of night sleep, shorter duration of daytime napping, less social jetlag, greater night-melatonin and day–night melatonin contrasts, greater energy intake in the morning, lower protein intake, and higher physical activity level were all factors related to better circadian health (higher GCS). In addition, a better daily pattern of light exposure with higher amplitude, increased PR, a greater IS, and a greater CFI (see Table 1 for definitions) was also associated with better circadian health. To evaluate the potential impact of different lifestyles on the association between circadian health and obesity, we tested for possible interactions between those factors associated with both circadian health and BMI/body fat percentage (i.e., bedtime, night sleep duration, night-melatonin, day–night melatonin contrast, total morning intake, protein intake, average physical activity and day–night activity contrast, and chronotype).

**Table 4:**
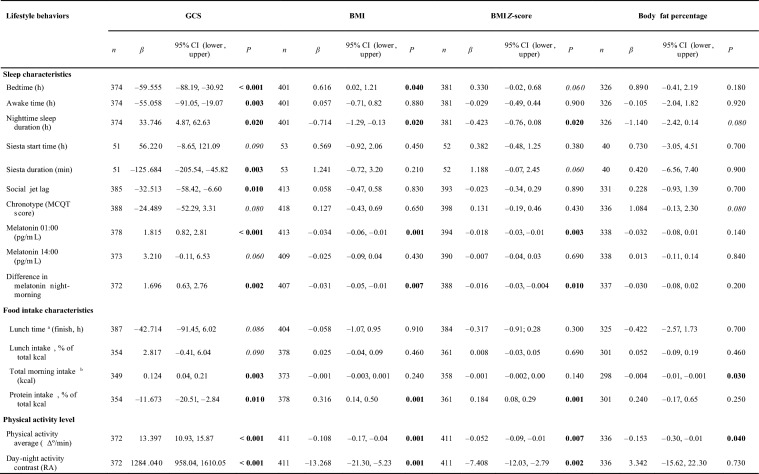

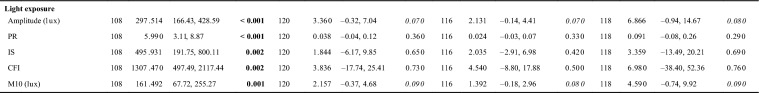
Association of lifestyle behaviors with GCS, BMI, BMI *Z*-score, and body fat percentage

Linear regression of lifestyle behaviors (independent variables) with GCS, BMI or body fat percentage as dependent variables. All models adjusted by sex, age, race, and academic year. We present only results of those lifestyle behaviors that were significant (*P* < 0.05) or borderline significant (*P* < 0.1), in the association with GCS. In bold are highlighted those significant results (*P* < 0.05), in italics it is showed borderline significant results (*P* < 0.1). *GCS* global circadian score, *BMI* body mass index, *MCQT* Munich chronotype questionnaire, *RA* relative amplitude, *PR* percentage of rhythmicity, *IS* interdaily stability, *CFI* circadian function index, *M10* average of measurements of the 10 consecutive hours with the maximum light exposure (log lux). ^a^Lunch timing, not included in the GCS; ^b^total morning intake calculated as: breakfast + midday + lunch energy intake

**Fig. 2:**
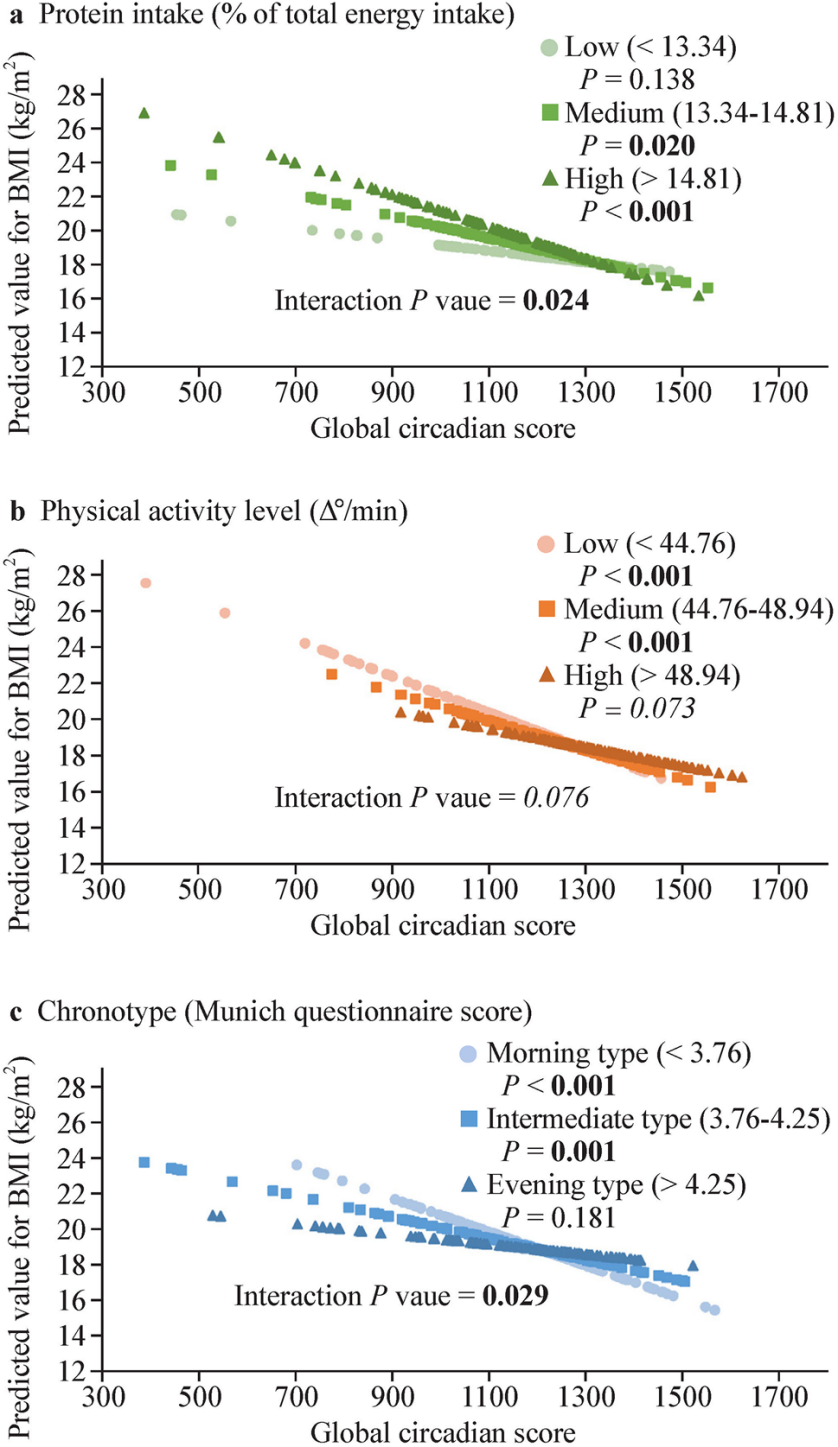
Lifestyle factor and global circadian score (GCS) interaction for body mass index (BMI). Predicted values of BMI according to tertile division of protein percentage of total energy intake (**a**), physical activity level (Δº/min) (**b**), and chronotype determined using the Munich chronotype questionnaire (MCTQ) score (**c**), plotted against the GCS. The predicted values for BMI (kg/m^2^) were calculated from the regression model after adjustment for age, sex, race, and academic year. Interaction *P* values indicate the statistical significance of the interaction term in the adjusted regression model (in bold, *P* < 0.05; in italics, *P* < 0.1). *P* values of each tertile indicate the statistical significance of the regression model between GCS and BMI within each tertile group for the protein percentage of total energy intake (**a**), physical activity level (Δº/min) (**b**), and chronotype determined using the MCTQ score (**c**)

Figure 2 shows the significant results of the lifestyle–GCS interactions for BMI. When the protein intake was categorized into high, medium, and low protein intake groups based on tertiles of the protein percentage of total energy intake (measured in kcal), we identified differential effects across the three groups (interaction* P* = 0.024). For the low-protein intake group (represented by light gray circles), no significant association was found between circadian health and obesity (*P* = 0.138). In contrast, in the medium (dark gray squares) and high protein intake groups (black triangles), a higher BMI was associated with poorer circadian health (*P* < 0.05).

Similarly, we identified differential effects across the three groups when children were categorized into high, medium, and low physical activity levels (interaction* P* = 0.076). For the high physical activity level group, no significant association was found between circadian health and obesity (*P* = 0.073), while significant associations were found between the medium and low activity level groups and higher BMI in children with poorer circadian health (*P* < 0.05).

Finally, we also identified significant differential effects across the individual chronotypes categorized into more morning, intermediate, and evening according to tertiles of Munich score (from the MCTQ; interaction* P* = 0.029). There was a significant association between circadian health and BMI in the morning and intermediate chronotypes (higher BMI with poorer circadian health, *P* < 0.05), while there was no significant association of circadian health with BMI in evening types (*P* = 0.181; Fig. 2).

## Discussion

Traditionally used techniques for assessing circadian health are self-report questionnaires, which can be biased since they are subjective and depend on good recall and the subject’s ability to complete them correctly and honestly [29]. In recent years, non-invasive, more objective, and thus more accurate methods have been developed to assess circadian-related rhythms in children [13]. The rapid development of wearable devices has provided a more objective approach. Moreover, algorithms that derive metrics to quantify the robustness, phase, and stability of rhythms have been developed to search for new biomarkers of circadian health [30]. Nevertheless, these new tools and algorithms have yet to be used, especially in pediatric populations.

The current study demonstrated that the developed GCS [11], which integrates objective measures such as TAP and cortisol concentrations with meal timing, is easy to use, versatile, and non-invasive [11] and is a valuable tool for preventing and managing obesity through detecting poor circadian health and consequent intervention. Furthermore, this approach follows the recommended methods for identifying circadian-related effects for research or clinical practice [7]. Indeed, our novel GCS captured obesity in this population of school-age children. Those children with obesity and those with abdominal obesity had 3.54 and 2.39 greater odds of having poor circadian health, than did those with a normal weight or with low waist circumference, respectively.

Among all the variables included in the GCS, the variables most strongly associated with obesity were the PR, a marker of the prominence/robustness of the TAP rhythm [31]; amplitude, which shows the difference between day and night TAP values; and IS, which determines the constancy of the 24-hour rhythmic pattern over days. These results are consistent with previous studies showing that PR is inversely correlated with obesity and ghrelin levels [32], the hormone that induces appetite. On the other hand, the amplitude of daily rest–activity patterns is inversely associated with adiposity and cardiometabolic risk in a previous study performed in teenagers [33]. Similarly, in adults, lower values of amplitude at the wrist temperature ultradian peak (from a 12-hour rhythm) have been associated with higher metabolic syndrome risk and ghrelin [32]. In addition, in the current study, a low IS in TAP was related to a higher BMI. A low IS has been previously associated with a worse lipid profile and is considered a relevant cardiometabolic risk factor in adults [34, 35].

In general, in the current population of school-aged children, obesity is related to worse circadian health and a significantly lower GCS. We and others have previously shown that obesity is strongly associated with circadian-related alterations in adults [36–38]. Here, we have demonstrated that this is also true for school-age children. Interestingly, children with healthy lifestyle habits were more likely to have better circadian health (measured by the GCS) and less obesity, while the opposite was also true. For example, a higher energy intake in the morning was associated with better circadian health and a lower body fat percentage in our pediatric population. Previous studies have shown that eating breakfast (vs. not eating it) reduces the risk of obesity in children and adolescents [39]. Furthermore, our study revealed that higher protein intake was associated with poorer circadian health and higher BMI. A recent meta-analysis [40] showed that children with higher protein intake (from animal sources) were more likely to have a higher BMI. In addition, we found that protein intake per day interacted with the GCS score for the individual BMI, suggesting the potential influence of protein intake on the association between circadian health and obesity.

Similar interactions were obtained for physical activity. We found a significant association between higher BMI and poorer circadian health in children with low-medium activity levels, while in the higher activity group, this association was not detected; indeed, in the high-level physical activity group, the association between the physical activity average (log) and lower BMI was independent of the circadian health score (beta =  – 24.71 kg/m^2^; *P* < 0.05), while this was not true for the other two groups (*P* > 0.05). Physical activity is considered the most modifiable factor of energy expenditure in school-age children, and higher physical activity levels are associated with lower BMI and percentage of body fat [41]. Furthermore, in our study, a greater contrast in day–night physical activity was associated with better circadian health and lower BMI. The day–night physical activity contrast is considered a marker of circadian-related alterations since a lower contrast is related to an impaired circadian system in young adults [42].

We also found a significant association between higher light exposure (average) and better daily patterns of light (higher amplitude, PR, IS, and CFI) and better circadian health (higher GCS). Previous studies have shown that a low amplitude of daily light in children, accompanied by a low day/night contrast in light due to higher light exposure during the evening/night, is associated with an impaired circadian system and melatonin secretion [43], as high light at night prevents the endogenous synthesis of melatonin by the pineal gland and reduces melatonin concentrations [44]. Melatonin is considered to be a sleep hormone because it is produced and secreted at night and is synthesized at a minimal level during the day [44]. In the present study, children with a low day/night contrast in melatonin had poorer circadian health and were more likely to have obesity. We previously reported that melatonin levels at 01:00 were lower in more evening-type children than in more morning-types, which could be related to circadian-related alterations [17] or poor circadian health.

Relatedly, in the current population, a higher Munich chronotype score, corresponding to a more evening type, tended to be associated with poorer circadian health (lower GCS) and a greater body fat percentage (both *P* < 0.1). We also found that those morning-type or intermediate-type children who had poorer circadian health (lower GCS), were more likely to have a higher BMI. However, those children with more evening chronotypes did not have a greater risk of obesity when they had poor circadian health. Our previous results showed that evening types, objectively determined as a higher value of TAP acrophase, were associated with higher BMI and metabolic alterations [17]. This finding suggested that the evening chronotype per se could be strongly associated with obesity, independent of circadian health.

In the discussion of our results, we need to consider several limitations. The cross-sectional design does not allow us to make inferences about the directionality or causality of the associations. We did not correct for multiple comparisons because all our outcomes are interrelated and the GCS comprises only one test of the relationship between obesity and circadian health. Therefore, future studies are needed to confirm our findings. Furthermore, while this GCS is based on objective and non-invasive measures, it may be challenging to be used in large-scale trials where saliva sampling is unavailable.

In conclusion, this study represents a significant step toward understanding novel aspects of the relationship between circadian health and obesity in school-age children. We demonstrated that the proposed GCS is a non-invasive and objective tool for detecting alterations in circadian health related to obesity (chronobesity). Due to its non-invasive nature, this score could be helpful in pediatric clinical practice. Emphasis should be placed on dietary intake (protein intake), physical activity, and individual chronotype, among other factors, to reduce the risk of obesity and improve overall health in children because these lifestyle factors might be involved in chrono-obesity. However, further intervention studies are needed to evaluate the effectiveness of behavioral interventions in modifying the GCS to reduce obesity.

## Supplementary Information

Below is the link to the electronic supplementary material.

Supplementary file1 (PDF 111 KB)

Supplementary file2 (PDF 254 KB)

Supplementary file3 (MPG 145035 KB)

## Data Availability

The datasets generated and analyzed during the current study are available in the Figshare repository (https://figshare.com/articles/dataset/Database_of_Global_Circadian_Score_in_Children/23717043).
