# Supplementary material for: Children with obesity have poorer circadian health as assessed by a global circadian health score
Source: World J Pediatr. 2024 Jun 8;20(8):787–800. doi: 10.1007/s12519-024-00804-3 (PMC11402851; doi:10.1007/s12519-024-00804-3)
Supplement: Supplementary file 1 — Supplementary file1 (PDF 111 KB) [file 12519_2024_804_MOESM1_ESM.pdf]

Supplementary Table 1. Formula (and its factors) used to determine the global circadian score for each subject studied

**GCS formula**	$GCS=(F1_{score}\times F1_{\%variance})+(F2_{score}\times F2_{\%variance})+(F3_{score}\times F3_{\%variance})+(F4_{score}\times F4_{\%variance})+(F5_{score}\times F5_{\%variance})$
**Factor scores**	
Factor 1 (F1 score)	$TAP_{CFI}\times0.96+TAP_{amplitude}\times0.96+TAP_{PR}\times0.92+TAP_{IS}\times0.92+TAP_{RA}\times0.89$
Factor 2 (F2 score)	$TAP_{mesor}\times0.95+TAP_{average}\times0.95$
Factor 3 (F3 score)	Cortisol 23:00×0.91+Log morning/evening cortisol ratio×(-0.89)+Cortisol 14:00×0.71
Factor 4 (F4 score)	Dinner start time×0.95+Dinner finish time×0.93
Factor 5 (F5 score)	Breakfast start time×0.95+breakfast finish time×0.92
**Factor variance**	
Factor 1 (F1 %variance)	16.96%
Factor 2 (F2 %variance)	10.42%
Factor 3 (F3 %variance)	8.42%
Factor 4 (F4 %variance)	7.86%
Factor (F4 %variance)	6.12%

Each factor score composed of each variable multiplied by its eigenvalue. Then, each factor score is multiplied by the percentage of the variance of the total information explained by this factor. *GCS* global circadian score, *TAP* integrative variable combining temperature, activity and position, *CFI* circadian function index, *PR* percentage of rhythmicity, *IS* interdaily stability, *RA* relative amplitude
