# Supplementary material for: Children with obesity have poorer circadian health as assessed by a global circadian health score
Source: World J Pediatr. 2024 Jun 8;20(8):787–800. doi: 10.1007/s12519-024-00804-3 (PMC11402851; doi:10.1007/s12519-024-00804-3)
Supplement: Supplementary file 2 — Supplementary file2 (PDF 254 KB) [file 12519_2024_804_MOESM2_ESM.pdf]

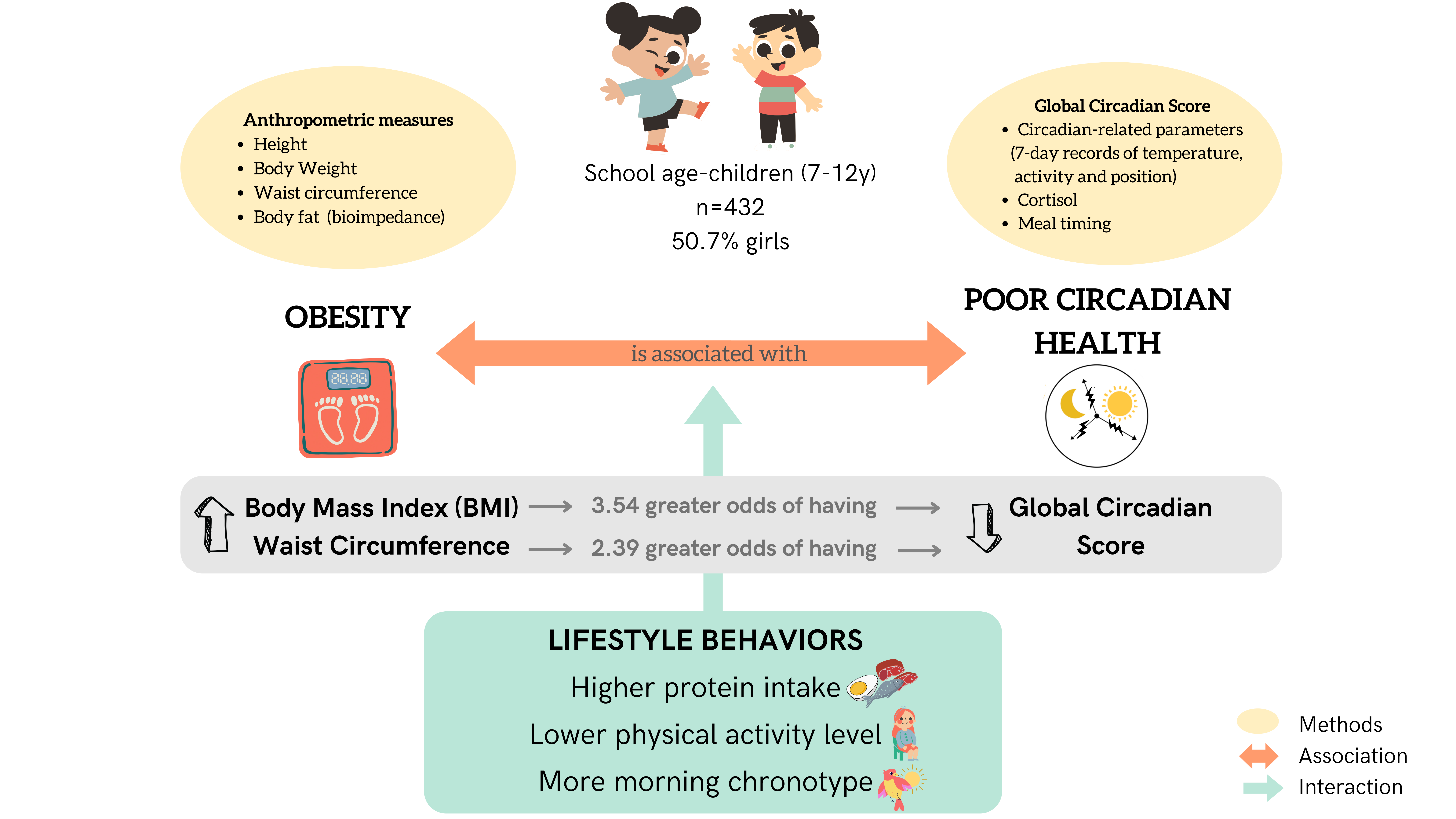
Anthropometric measures
Height
Body Weight
Waist circumference
Body fat (bioimpedance)
School age-children (7-12y)
n=432
50.7% girls
Global Circadian Score
Circadian-related parameters (7-day records of temperature, activity and position)
Cortisol
Meal timing
OBESITY
is associated with
POOR CIRCADIAN HEALTH
Body Mass Index (BMI) → 3.54 greater odds of having →
Waist Circumference → 2.39 greater odds of having →
Global Circadian Score
LIFESTYLE BEHAVIORS
Higher protein intake
Lower physical activity level
More morning chronotype
Methods
Association
Interaction
